# How to use meropenem in pediatric patients undergoing CKRT? Integrated meropenem pharmacokinetic model for critically ill children

**DOI:** 10.1128/aac.01729-23

**Published:** 2024-04-24

**Authors:** Laura Butragueño-Laiseca, Iñaki F. Troconiz, Santiago Grau, Nuria Campillo, Belén Padilla, Sarah Nicole Fernández, María Slöcker, Laura Herrera, María José Santiago

**Affiliations:** 1 Pediatric Intensive Care Unit, Hospital General Universitario Gregorio Marañón, Madrid, Spain; 2 Gregorio Marañón Health Research Institute (IISGM), Madrid, Spain; 3 Pediatrics Department, Universidad Complutense de Madrid, Madrid, Spain; 4 Primary Care Interventions to Prevent Maternal and Child Chronic Diseases of Perinatal and Development Origin Network (RICORS) RD21/0012/0011, Carlos III Health Institute, Madrid, Spain; 5 Pharmacometrics and Systems Pharmacology Research Unit, Department of Pharmaceutical Sciences, School of Pharmacy and Nutrition, University of Navarra, Pamplona, Spain; 6 IdiSNA, Navarra Institute for Health Research, Pamplona, Spain; 7 Pharmacy Department, Hospital del Mar, Universitat Autònoma de Barcelona, Barcelona, Spain; 8 Clinical Microbiology Department, Hospital General Universitario Gregorio Marañón, Madrid, Spain; University Children's Hospital Münster, Münster, Germany

**Keywords:** meropenem, acute kidney injury, population pharmacokinetics, critically ill children, dose individualization, continuous kidney replacement therapy, continuous renal replacement therapy

## Abstract

Standard dosing could fail to achieve adequate systemic concentrations in ICU children or may lead to toxicity in children with acute kidney injury. The population pharmacokinetic analysis was used to simultaneously analyze all available data (plasma, prefilter, postfilter, effluent, and urine concentrations) and provide the pharmacokinetic characteristics of meropenem. The probability of target fT > MIC attainment, avoiding toxic levels, during the entire dosing interval was estimated by simulation of different intermittent and continuous infusions in the studied population. A total of 16 critically ill children treated with meropenem were included, with 7 of them undergoing continuous kidney replacement therapy (CKRT). Only 33% of children without CKRT achieved 90% of the time when the free drug concentration exceeded the minimum inhibitory concentration (%fT > MIC) for an MIC of 2 mg/L. In dose simulations, only continuous infusions (60–120 mg/kg in a 24-h infusion) reached the objective in patients <30 kg. In patients undergoing CKRT, the currently used schedule (40 mg/kg/12 h from day 2 in a short infusion of 30 min) was clearly insufficient in patients <30 kg. Keeping the dose to 40 mg/kg q8h without applying renal adjustment and extended infusions (40 mg/kg in 3- or 4-h infusion every 12 h) was sufficient to reach 90% fT > MIC (>2 mg/L) in patients >10 kg. In patients <10 kg, only continuous infusions reached the objective. In patients >30 kg, 60 mg/kg in a 24-h infusion is sufficient and avoids toxicity. This population model could help with an individualized dosing approach that needs to be adopted in critically ill pediatric patients. Critically ill patients subjected to or not to CKRT may benefit from the administration of meropenem in an extended or continuous infusion.

## INTRODUCTION

Meropenem is commonly prescribed in critically ill children for targeted therapy in severe and multidrug-resistant bacterial infections. Meropenem, as a carbapenem, has a time-dependent bactericidal effect. Optimal dosing is evaluated based on the ability to maintain meropenem concentrations in plasma above the minimum inhibitory concentration (MIC) of the infecting pathogen throughout the dosing interval (fT > MIC) (1, 2). However, the optimal clinical pharmacokinetic/pharmacodynamic (PK/PD) target remains uncertain as it varies from 50% fT > MIC to 100% fT > 4× MIC. The latter would be desirable for critically ill patients (3, 4)

Therapeutic drug monitoring (TDM) has been demonstrated to optimize meropenem levels and PK/PD targets in adult patients (1). The same is necessary for children. However, pharmacokinetic studies to guide meropenem dosing in critically ill children are still scarce (5–9). Development during childhood is associated with changes in kidney function, which affects renal clearance. Other pharmacokinetic processes such as absorption and non-renal metabolism may be also altered (10). In addition to age-related differences in pharmacokinetics, the critically ill patient suffers several pathophysiologic changes: an increased apparent volume of distribution (*V*) due to fluid balance strategies and intravascular perfusion changes; decreased or increased cardiac output due to sepsis that can result indysregulation of renal blood flow and drug clearance (CL), in addition to inflammatory response syndrome, capillary leak syndrome, and hypoalbuminemia. All these comorbidities are associated with changes in the PK of antibiotics (11)

Acute kidney injury (AKI) is extremely common in children admitted to pediatric intensive care units (up to 25% of patients) and 10%–15% will need continuous kidney replacement therapy (CKRT) (12). Infections are common in patients on CKRT (13), and multidrug-resistant bacterial infections are increasing (14), posing a real threat to critically ill children. In this setting, antibiotic dosing in critically ill patients with AKI or undergoing CKRT is challenging. Limited data are available regarding meropenem PK in children and adolescents with AKI, and, to our knowledge, there is only one previous study (5) including data about meropenem in pediatric patients on CKRT.

The aim of this study was to characterize the PK of meropenem in critically ill pediatric patients with and without CKRT. For this purpose, a population pharmacokinetic model in 16 critically ill children treated with meropenem, with 7 of them undergoing CKRT, was developed.

## MATERIALS AND METHODS

The results of this prospective study are part of a broader project intended to describe the PK properties of several antibiotics in pediatric patients with CKRT (European Regional Development Fund, ref. RD16/0022/0007). All patients receiving antibiotics and with an intravenous catheter allowing needle-free blood draws in the PICU were eligible for this prospective study.

### Dosing, sampling, and analytical method

According to the hospital protocol, patients received 40 mg meropenem/kg of body weight every 8 h intravenously. At the fourth dose, in patients with CKRT, the dosing interval was increased to 12 h, following the recommended renal adjustment (15). No patients in the no-CKRT group required adjustment based on renal function. Blood samples were obtained through an intravenous catheter allowing needle-free blood draws in the pediatric intensive care unit. Sampling began once patients had received at least three doses of meropenem. One milliliter blood and urine samples were collected in heparinized tubes before (T0) and at 2, 4, 6, and 8 h after starting the infusion (T2, T4, T6, and T8). In patients with CKRT, blood samples from the prefilter and postfilter ports and a sample from the effluent port of the Prismaflex (Baxter Int.) device were drawn simultaneously. There were 18 samples in each CKRT patient. In no-CKRT patients, six blood samples and six urine samples were collected. Blood samples were centrifuged at 2,500 rpm for 10 min to yield at least 350 mcL of plasma and then stored at −80°C.

Meropenem concentrations were quantified using a validated high-performance liquid chromatography (HPLC) method. HPLC system was an Alliance e2695 with UV detection W2489 (Waters). Piperacillin standard (Sigma) and quality controls (Teknokroma-Cromsystems) were used for calibration curves and quality controls, respectively. Methanol, acetonitrile, water chromatography, and ammonium acetate were purchased from Merck and PanReac.

Protein precipitation of plasma and urine samples was performed with methanol 1/2 (vol/vol%). The urine samples were diluted before precipitation 1/20 or 1/50 (vol/vol%). The separation was performed on a Symmetry C18 column (100 × 4.6, 3.5 µm) with UV detection set at 254 nm. The mobile phase consisted of ammonium acetate buffer: acetonitrile (70:30), delivered at 1.2 mL/min. Linearity was established over the concentration range from 0.5 to 1,000 mg/L. Calibration curve determination coefficients were ≥0.999, and response factors CV% < 15%. The lower limit of quantification (LOQ) was established at 0.5 mg/L. Accuracy was <11%, and precision, expressed as coefficient of variation, was always below 10%.

### Pharmacokinetic analysis

The population pharmacokinetic analysis was used to simultaneously analyze all available data [plasma (*C*_*P*_), prefilter (*C*_Pre_), postfilter (*C*_post_), effluent (*C*_Effl_), and urine (*C*_Ur_) concentrations]. The analyses were performed using the software NONMEM 7.4 (16) with the first order conditional estimation method and the INTERACTION option.

Meropenem concentration data were logarithmically transformed for the analysis. Interpatient variability (IPV) was modeled using the exponential model, and the residual error was characterized with an additive model on the logarithmic scale. Different magnitudes of residual error were estimated for each of the different types of measured concentrations.

Model selection was guided by the minimum value of the objective function, parameter precision, and visual inspection of the goodness-of-fit plots. A reduction of 3.84 and 6.61 points in the minimum value of the objective function [approximately equal to −2 × Log (likelihood) (−2LL)] between two hierarchical models differing in one parameter was considered significant at the 5% and 1% levels of significance, respectively. The precision of model parameters was evaluated by calculating the coefficient of variation as the ratio between the standard error and the estimate of the parameter and multiplied by 100.

#### Model building

Model building started with the development of the base population model, followed by the selection of covariates and then by model evaluation.

##### Base population model

Disposition of meropenem in the body was described with compartmental models integrating into the same model structure *C*_*P*_ and *C*_Pre_, *C*_post_, *C*_Effl_, and urine *C*_Ur_ based on the approach suggested by Broeker et al. (17). The following set of ordinary differential equations corresponds to a two compartments model (although during the analysis, the one- and three-compartment models were also considered):


(1) $$\frac{dA_{P,Pre}}{dt}=\frac{{CL}_{D}}{V_{2}}\times A_{2}-\frac{{CL}_{D}}{V_{1}}\times A_{P,Pre}-\frac{\left({CL}_{Renal}+{CL}_{RRT}+{CL}_{M}\right)}{V_{1}}\times A_{P,Pre}$$

(2) $$\frac{dA_{2}}{dt}=\frac{{CL}_{D}}{V_{1}}\times A_{P,Pre}-\frac{{CL}_{D}}{V_{2}}\times A_{2}$$

(3) $$\frac{dA_{Ur}}{dt}=\frac{{CL}_{Renal}}{V_{1}}\times A_{P,Pre}$$

where *A*_*P*,Pre_ represents the amount of meropenem in the central compartment (including plasma) and the prefilter, *A*_2_ in the peripheral compartment, and *A*_UR_ in urine. *V*_1_ and *V*_2_ are the apparent volumes of distribution of the central and peripheral compartments, respectively, and CL_*D*_ is the distribution clearance. Total elimination clearance (CL) is the sum of the renal (CL_Renal_), metabolic (CL_*M*_) and renal replacement therapy (CL_CKRT_). In the current analysis, the use of drug concentration measurements in plasma, prefilter, postfilter, effluent, and urine allowed for the estimation of CL_Renal_, CL_*M*_, and CL_RRT_. CL_CRT_ was absent in patients without hemofilters.

Predicted *C*_*P*_ and *C*_Pre_ were obtained as *A*_1_/*V*_1_. *C*_Ur_ was obtained as *A*_Ur_/*U*_Vol_, where *U*_Vol_ is the measured volume of urine excreted in each urine recovery interval.

The parameter CL_CKRT_ links together *C*_Pre_ with *C*_Post_ and *C*_Effl_ as represented by equations 4 and 5 (17):


(4) $$C_{Post}=C_{Pre}\times \left(1-\frac{CL_{RRT}}{\varphi_{Pl,corr}}\right)$$

(5) $$C_{Effl}=\frac{CL_{RRT}\times C_{Pre}}{\varphi_{Effl}}$$

where $\varphi_{Pl,corr}$ and $\varphi_{Effl}$ are the corrected plasma and total effluent flows. The value of $\varphi_{Pl,corr}$ was calculated as $\varphi_{Blood}$ × BPR, $\varphi_{Pl,corr}$ being the blood flow rate and BPR the blood to plasma concentration ratio, an additional parameter to be estimated from the model. The values of $\varphi_{Blood}\;and\;\varphi_{Effluent}$ were measured during the course of the study.

The existence of reversible and irreversible interaction mechanisms between meropenem and filter membrane of the hemofilter was explored, as well as the significance of the diagonal and off-diagonal elements of the Ω variance-covariance matrix. Different magnitudes of residual error were estimated for each of the different types of measured entities.

##### Covariate selection

The stepwise covariate model (SCM) building approach was used to select the significant covariates (18). The selection process starts with a forward selection followed by a backward deletion procedure with default levels of significance of 0.05 and 0.01, respectively. Both linear and different non-linear relationships between the continuous covariates and model parameters were evaluated.

The following covariates were tested in all pharmacokinetic parameters associated with interindividual variability: height, age, body weight (WGT), albumin concentration, estimated glomerular filtration rate (eGFR), patient type (with or without CKRT), and surface area of the hemofilter. The eGFR was estimated using Cystatin C (Chronic Kidney Disease Epidemiology Collaboration [CKD-EPI] Equation) and Schwartz formula and integrated into the model for those patients without CKRT (19–21). Continuous covariates were entered into the model normalized by the corresponding median value of the patient population.

##### Model evaluation

The selected model was evaluated using the simulation-based diagnostic prediction-corrected visual predictive check (VPC) (22) generated as follows: 1,000 data sets with the same study characteristics as the original one were simulated. For each simulated data set, time bin and concentration type, the 5th, 50th, and 95th percentiles, of the simulated concentrations were calculated, and their corresponding 90% confidence intervals were plotted together with the 5th, 50th, and 95th percentiles obtained from the raw data. Parameter precision was further evaluated using sampling importance resampling (SIRS) (23).

### Probability of target attainment

The developed model was used to evaluate the target attainment (defined as the percentage of time of plasma concentrations above MIC, %TA). Total simulated plasma concentrations were used to calculate target attainment since the unbound fraction of meropenem in plasma is close to 1 [0.98 (24, 25)]. The target MIC values were 0.25, 0.5, 1,2, 4, 8, and 18 mg/L. One thousand patients with or without CKRT were simulated per weight group (3–10, 10–30, and 30–60 kg) and dosing regimen. The mean %TA for each dosing regimen during the first administration of the second day of treatment was calculated.

A correlation between age and body weight, but not with eGFR, was found using data from patients in different studies. Therefore, the simulations aforementioned were done taking into account that correlation. Figure S1 shows simulated weight vs age pairs together with raw data.

In CKRT subjects, the weight groups of 3–10, 10–30, and 30–60 kg were assigned to low (0.2 m^2^), middle (0.6 m^2^), and high filter (1.2 m^2^) surfaces, respectively, while the same weight groups in non-CKRT subjects were assigned GFR values of 60–120, 60–120, and 120–130 mL/min/1.73 m^2^, respectively.

The 95% prediction intervals of the simulated plasma concentration per patient type, weight group, and dosing schedule were computed and graphically represented together with the toxicity thresholds of 45 and 65 mg/L (26–28).

### Software

Data processing and exploratory analyses of the raw data, model selection, model evaluation, and simulation plotting were carried out with R 4.0.2 (29) and RStudio 1.3.1073 (30). NONMEM 7.4 along with PsN 4.9 (31) were used to perform the analysis and the simulation-based diagnostics such as prediction-corrected VPCs, SCM, and SIRS analysis.

## RESULTS

### Demographics and clinical data

The model was developed with data obtained from 16 critically ill children treated with meropenem, with 7 of them undergoing CKRT. In Table 1, a summary of patient characteristics at baseline is presented. Most patients (62.5%) were in the postoperative period of congenital cardiopathy. In the CKRT group, the median age was 48 (IQR 5–106) months, and the weight was 20 (IQR 7.4–40) kg. In the group without CKRT, the median age was 8 (IQR 3.5–82) months and weight 7.5 (IQR 5.5–17.5) kg, but these differences were not statistically significant. Patient’s severity was estimated using the PRISM III score (32). The PRISM score was higher in CKRT patients (*P* = 0.026). In the group without CKRT, the median estimated glomerular filtration rate was 89 (IQR 69–122) and urine output was 1,100 (719–1,150) mL. There was a median of residual diuresis in the CKRT group of 141 (52–457) mL. In the main diagnosis given in the supplemental material (Table S1), suspected infection, isolated bacteria, and MIC (mg/L) (when isolated) are listed. Tables 2 and 3 show the median hemofilter parameters and size, mean running time, and effluent flow of the filters. Prismaflex (Baxter Int.) CKRT devices were used. Polyacrylonitrile AN69 hollow-fiber hemofilters were used depending on the body surface area of the patient and on the pump employed. HF20 0.2 m^2^, 58 mL (Baxter Int.) was used in children weighing less than 10 kg; M60 (Baxter Int.) 0.6 m^2^, 93 mL was used in patients weighing between 10 and 30 kg, and M100 (Baxter Int.) 0.9 m^2^, 152 mL in children weighing over 30 kg. Commercially prepared bicarbonate-buffered hemofiltration replacement and dialysis fluids were used. Anticoagulation of the circuit was performed with citrate in four patients and with a continuous heparin infusion in three patients. The total median ultrafiltration rate was 1,253 (335–1,870) mL/h. No patient experienced any adverse effect, including neurotoxicity or nephrotoxicity, associated with treatment.

**TABLE 1:** Summary of both group patient’s basal characteristics^*a*^

	Patients without AKI median (IQR)	Patients with CKRT median (IQR)	*P*
Age (months)	8 (3.5–82)	48 (5–106)	0.288
Weight (kg)	7.5 (5.5–17.5)	20 (7.4–40)	0.185
Corporal surface (m^2^)	0.38 (0.3–0.73)	0.79 (0.36–1.1)	0.204
eGFR	89 (69–122)	CKRT	N/A^*b*^
PRISM III score	5.5 (0–12.25)	15 (9.5–20)	0.026
Urine output (mL)	1,100 (719–1,150)	141 (52–457)	<0.01
PICU stay (days)	70 (17–145)	42 (23–94)	0.681
Hospital stay (days)	105 (62–193)	70 (48–105)	0.299

	Patients without AKI number (%)	Patients with CKRT number (%)	*P*
Diagnosis cardiopathy	8 (89)	4 (57)	0.262
Postoperatory period	6 (67)	5 (71)	0.635
ECMO	2 (22)	3 (43)	0.596
Mortality	2 (22)	4 (57)	0.302

^*a*^ Parameter estimates are represented together with the corresponding coefficient of variation.

^*b*^ N/A, not applicable.

**TABLE 2:** Hemofilter parameters of the seven patients treated with CKRT

Parameter	Median (IQR)
Blood flow (mL/min)	70 (40–100)
Anticoagulation citrate/heparin (number of patients)	4/3
Citrate dose (mmol/L)	2.8 (2.1–3.6)
Citrate flow (mL/h)	900 (680–1,125)
Substitution flow (mL/h)	50 (50–140)
Dialysis flow (mL/h)	400 (150–1,000)
Ultrafiltration rate (mL/h)	1,253 (335–1,870)
Extraction rate (mL/h)	80 (45–80)
Heparin dose (UI/kg/h)	20 (10–25)

**TABLE 3:** Number and type of filters used in the study

Filter	Filter surface (m^2^)	Number of filters	Mean running time (SD) (hours)	Mean effluent flow (SD) (mL/kg/h)
Low	0.2	3	65.6 (18)	44 (8)
Medium	0.6	2	84 (42)	60 (4)
High	1.2	2	53 (37)	46 (12)

### Population pharmacokinetic modeling

#### Brief description of the data available for the analysis

The number of concentrations of meropenem used to develop the population pharmacokinetic model was 212, of which 60% (*n* = 118) was obtained from patients under CKRT. The numbers of samples taken from plasma, postfilter, effluent, and urine was 99, 38, 26, and 49, respectively. None of them were reported below LOQ. The urine output in patients without CKRT ranged from 14 to 125 mL/h. Four patients under CKRT had anuria, and the other three had urine volumes ranging from 10 to 123 mL during the time interval of the pharmacokinetic curve.

#### Base population model

The one-compartment model described the data significantly worse than the two-compartment model (*P* < 0.001). When meropenem pharmacokinetics was attempted to be described with a three-compartment model, the fit was not improved significantly (*P* > 0.05) with respect to the two-compartment model and some model parameters were not identifiable. The simultaneous analysis of the different types of meropenem concentrations untangled the total elimination clearance into renal, non-renal (CLM), and the one representing the contribution of continuous renal replacement therapy (CLCKRT). The results indicate that the distribution of meropenem into the red blood cells was negligible, and the value of BPR shows a value equal to 1 hematocrit. Data supported the estimation of inter-individual variability associated with CLR, CLM, CLCKRT, and *V*, the apparent volume of distribution of the central compartment with values expressed as coefficient of variation of 115%, 81%, 91%, and 103%, respectively. Covariance between random effects was not significant (*P* > 0.05), and the value of residual variability of the different concentration entities ranged between 13% and 16%.

#### Selection of covariates

Renal function and maturation represented by eGFR and AGE, respectively, significantly influenced renal clearance in patients without CKRT (*P* < 0.01). The covariate model indicates that 50% of adult renal function is achieved at the age of 15.9 months and that an increment in eGFR of 10 mL/min/1.73 m^2^ is associated with a 10.5% increase in CLR. The Hill coefficient of the AGE function was not significantly different from 1 (*P* > 0.05). The value of CLR for a patient with normal and fully mature renal function is 5.5 L/h. For the three patients under CKRT with diuresis, CLR was 0.96 L/h (*P* < 0.01) and resulted independently from eGFR and AGE (*P* > 0.05). The surface area of the hemofilter impacted CLCKRT significantly (*P* < 0.01). The depurative efficiency of the extracorporeal elimination was reduced and augmented by 73% and 50%, respectively, for low and high surface area filters compared to middle size. Body weight shows significant covariate effects on *V*1 (*P* < 0.01) but did not affect any of the other model parameters (*P* > 0.05). Total protein or albumin serum levels, other demographics, and laboratory indexes did not impact model parameters. The apparent volume of distribution of the peripheral compartment, inter-compartmental clearance, and CLM were not influenced by the covariates investigated in the current evaluation (*P* > 0.05). The incorporation of the aforementioned covariate effects reduced the estimates of the inter-individual variability obtained from the base population to 30%, 86%, and 48% for CLR, CLM, and V1, respectively.

#### Model evaluation and exploration

**Fig 1:**
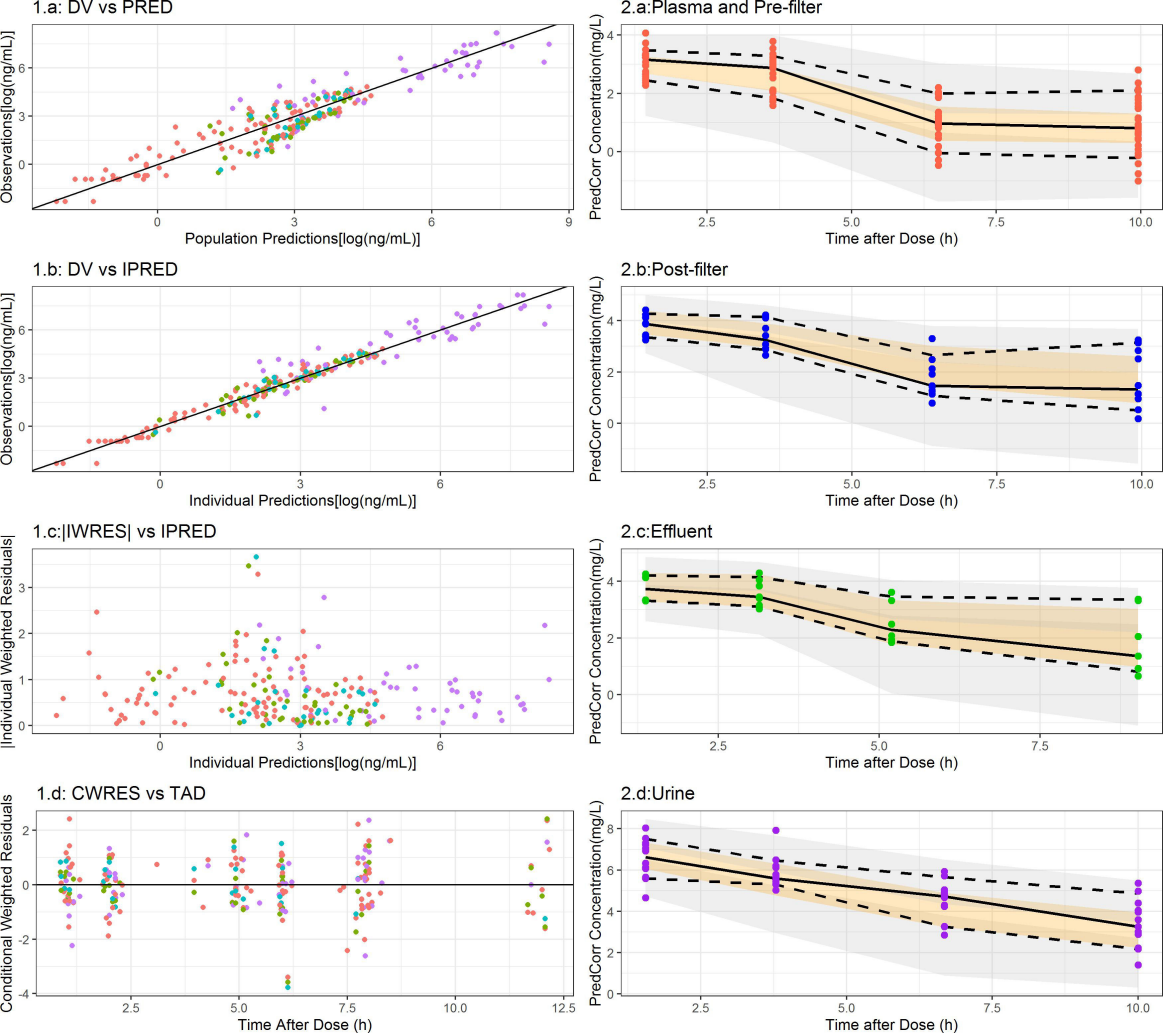
Left panels (1.a, b, c, d): goodness-of-fit plots. Red, blue, green, and purple solid circles represent concentrations of meropenem in plasma/prefilter, postfilter, effluent, and urine, respectively. 1.a: DV, dependent variable (observed measurements); PRED, typical population model predictions; 1.b: IPRED, individual model predictions; 1.c: *|*IWRES*|*, absolute individual weighted residuals; 1.d: CWRES, conditional weighted residuals; and TAD, time after dose. Solid lines represent the perfect fit. Right panels (2.a, b, c, d): population-corrected visual predictive checks. Colored solid circles, normalized observations. Solid and dashed lines represent the median and 2.5th and 97.5th percentiles, respectively, of the normalized observations. Colored areas cover the 95% prediction intervals of the 2.5th and 97.5th (gray) and 50th (red) percentiles obtained after simulating 1,000 thousand clinical studies.

**Fig 2:**
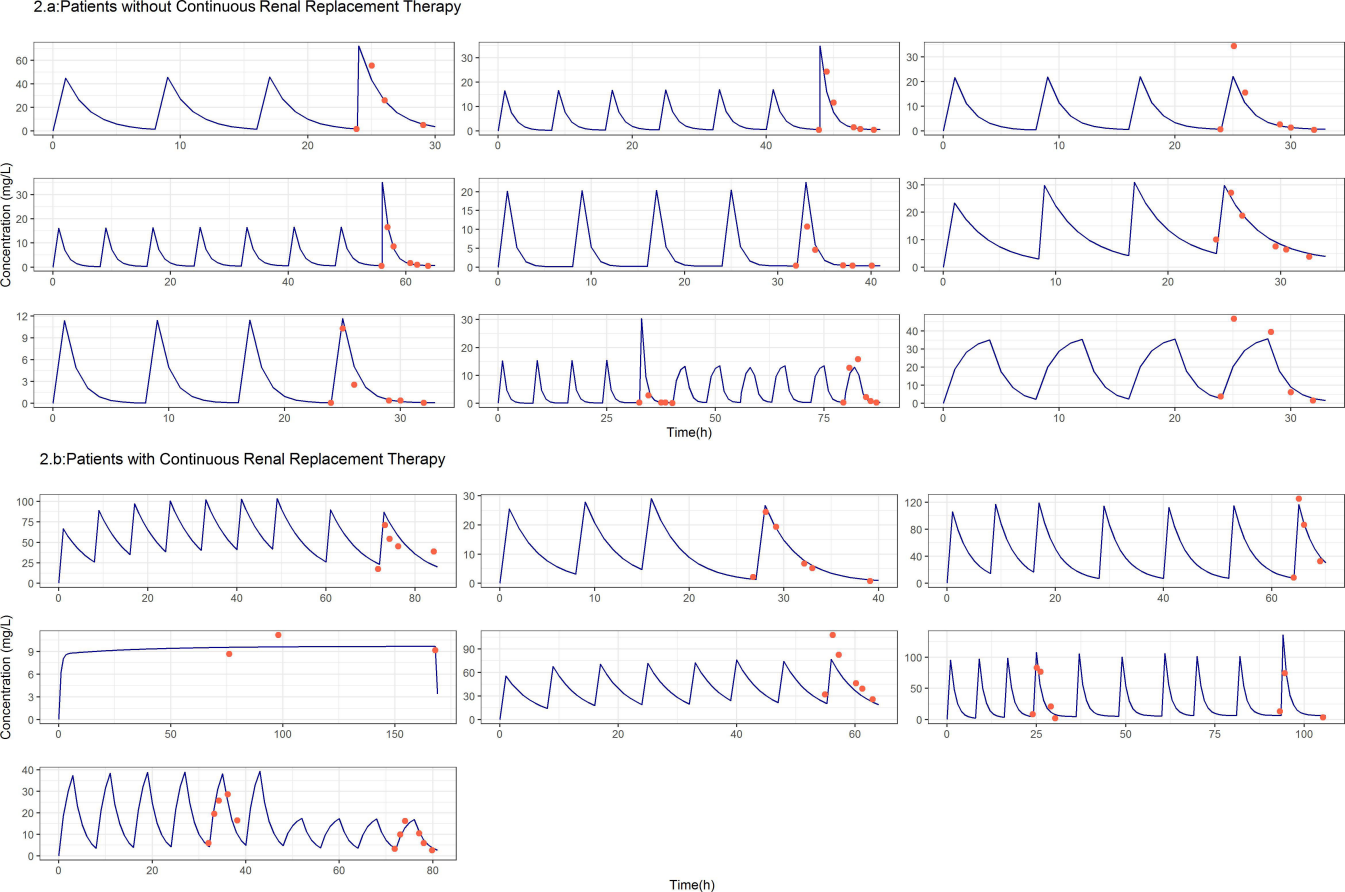
Individual model predictions. Each panel shows the (i) individual model predicted plasma concentration vs time profiles (blue) considering the full dosing scheme, and (ii) observed concentrations in red.

The estimates of the parameters corresponding to the selected model are listed in Table 4. The values of RSE (%) suggest that, in general, parameters were estimated precisely, a result that is supported by the SIRS analysis. For all model parameters (fixed and random effects), the lower limit of the 95% confidence intervals was greater than 0. The goodness-of-fit plots and the simulation-based diagnostics presented in Fig. 1 indicate an adequate characterization of the data. The full model predicted plasma pharmacokinetic vs time profiles from the start of treatment to the last follow-up time for all the patients studied is shown in Fig. 2 together with the observed concentration measurements. The numbers (%) of patients without CKRT with meropenem predicted plasma concentrations for at least 90% of the treatment period over the MIC values of 0.25, 1, 2, 4, 8, and 16 mg/L were 6 (66), 4 (44), 3 (33), 1 (11), 0 (0), and 0 (0), respectively. The corresponding values for the patients under CKRT were 7 (100), 7 (100), 6 (85), 5 (71), 4 (44), and 2 (22), respectively.

**TABLE 4:** Population pharmacokinetic parameter estimates of meropenem in critically ill children without and with continuous kidney replacement therapy^*a*^

Parameter	Parameter model	Estimates (RSE%) (95% CI)	IPV (RSE%)	Shrinkage (%)
CL_*R*_ (L/h)	$\theta_{CLR}\times \left(\frac{eGFR}{95}\right)\times \left(\frac{AGE}{AGE+\theta_{50}}\right)$	*θ*_CLR_= 5.5 (13) (4.4–4.8) *θ_50_* = 15.6 (16) (11.3–20)	30 (60) (12–51)	43
CL_*R*_CKRT_ (L/h)	$\theta_{CLR}$	*θ*_CLR_CKRT_ = 0.96 (53) (0.5–1.8)		
CL_CKRT_ (L/h)	*_ $\theta_{CLCKRT}\times \theta_{FILT}$ _*	*θ*_CLCKRT_ = 1.26 (19) (0.9–1.6) *θ*_FILT_Med_ = 1 (reference) *θ*_FILT_High_ = 1.5 (23) *θ*_FILT_Low_ = 0.27 (22)	NS	N/A
CL_*M*_ (L/h)	$\;\;\;\;\;\theta_{CLM}$	*θ*_CLM_ = 0.91 (25) (0.6–1.3)	86 (59) (60–122)	18
*V*_1_ (L)	$\;\theta_{V1}\times \frac{WGT}{10}$	*θ_V_* = 4.75 (17) (3.6–5.9)	48 (30) (33–62)	14
CL_*D*_ (L/h)	*_ $\theta_{CLD}$ _*	*θ*_CLD_ = 0.28 (25) (0.22–0.39)	NS	N/A
*V*_2_ (L)	$\theta_{V2}$	*θ*_VT_ = 10.7 (41) (6.6–17.6)	NS	N/A
Residual error [Log (mg/L)]	Plasma and prefilter Postfilter Effluent Urine	0.38 (12) (0.34–0.45) 0.38 (30) (0.31–0.52) 0.37 (36) (0.27–0.53) 0.86 (13) (0.7–1.1)	NS	9 6 4 3

^*a*^ RSE%, percentage relative standard error; CI, confidence interval obtained from the SIRS analysis; IPV, inter-patient variability expressed as CV (%) calculated as $\sqrt{e^{\omega^{2}}-1}\times 100$ , where *ω*^2^ is the variance; NS, not significant (*P* > 0.05); N/A, not applicable; CL_*R*_, CL_CKRT_, and CL_*M*_, renal, CKRT, and non-renal clearance, respectively; *V*_1_ and V_2_, apparent volumes of distribution of the central and peripheral compartments, respectively; CL_*D*_, inter-compartmental (distribution) clearance; eGFR, estimated glomerular filtration rate (median = 95 mL/min/1.73 m^2^); FILT_Low, Med, High filter surface 0.2, 0.6 (reference), and 1.2 m^2^, respectively; WGT, body weight (median = 10 kg).

**Fig 3:**
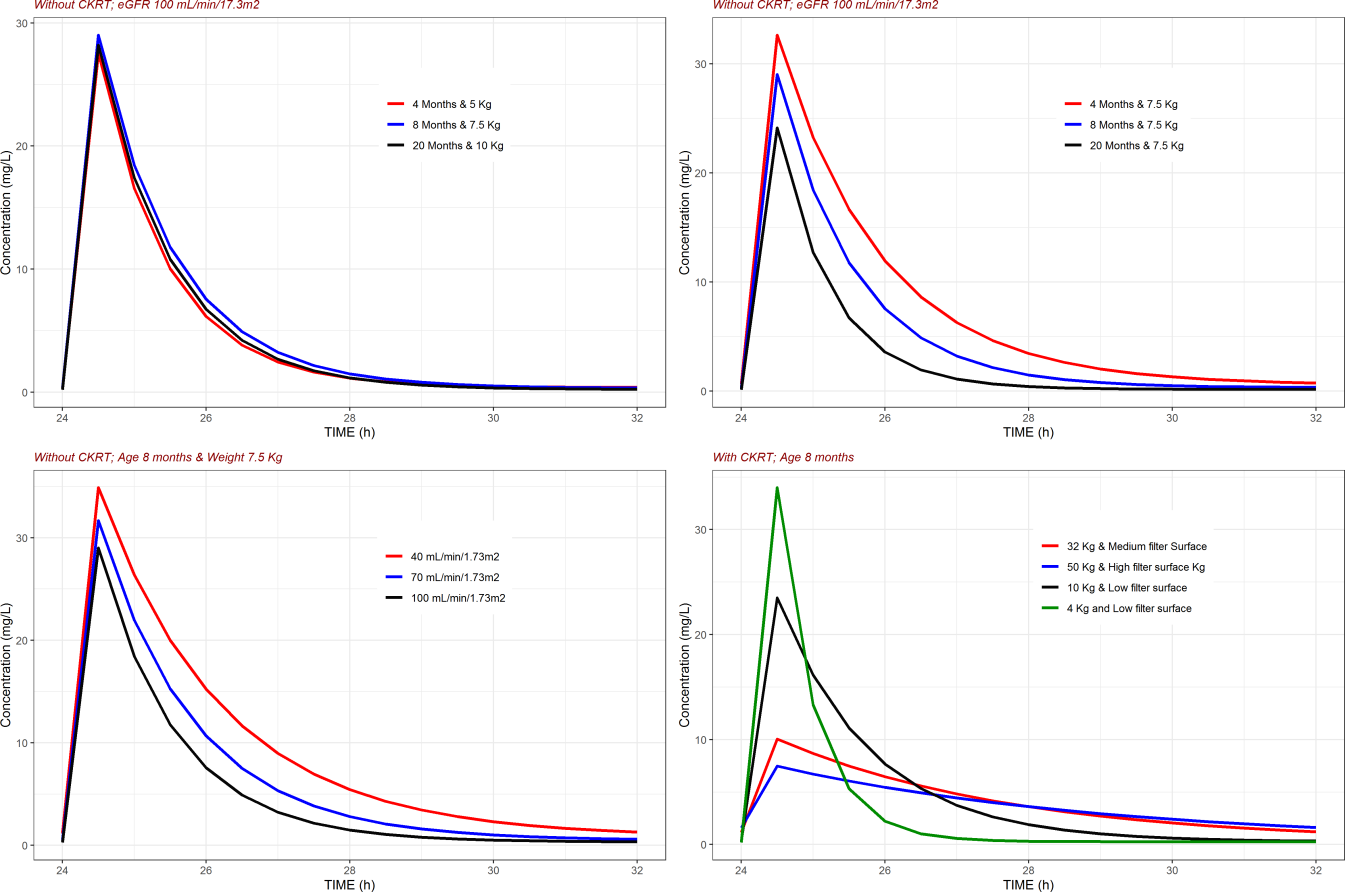
Impact of selected covariates on the typical plasma concentration vs time profiles of meropenem. Dosing regimen consisted of 150 mg of meropenem infused intravenously during 30 min every 8 h, except in the upper right panel, where the dose levels administered were 100, 150, and 200 mg for patients weighting 5, 7.5, and 10 kg, respectively.

Figure 3 shows the impact of the selected covariates on the typical plasma concentration vs time profiles of meropenem. The upper left panel shows that for a patient with normal kidney function, body weight dose adjustments result in very similar concentration vs time profiles. An increase from 4 to 8 months of age (upper right panel) or from 40 to 100 mL/min/1.73m^2^ in eGFR (lower left panel) is associated with a marked reduction in systemic exposure. Finally, the lower right panel shows how body weight and filter size have an impact on maximum plasma levels and area under the plasma vs time concentration curve.

### Probability of target attainment

**Fig 4:**
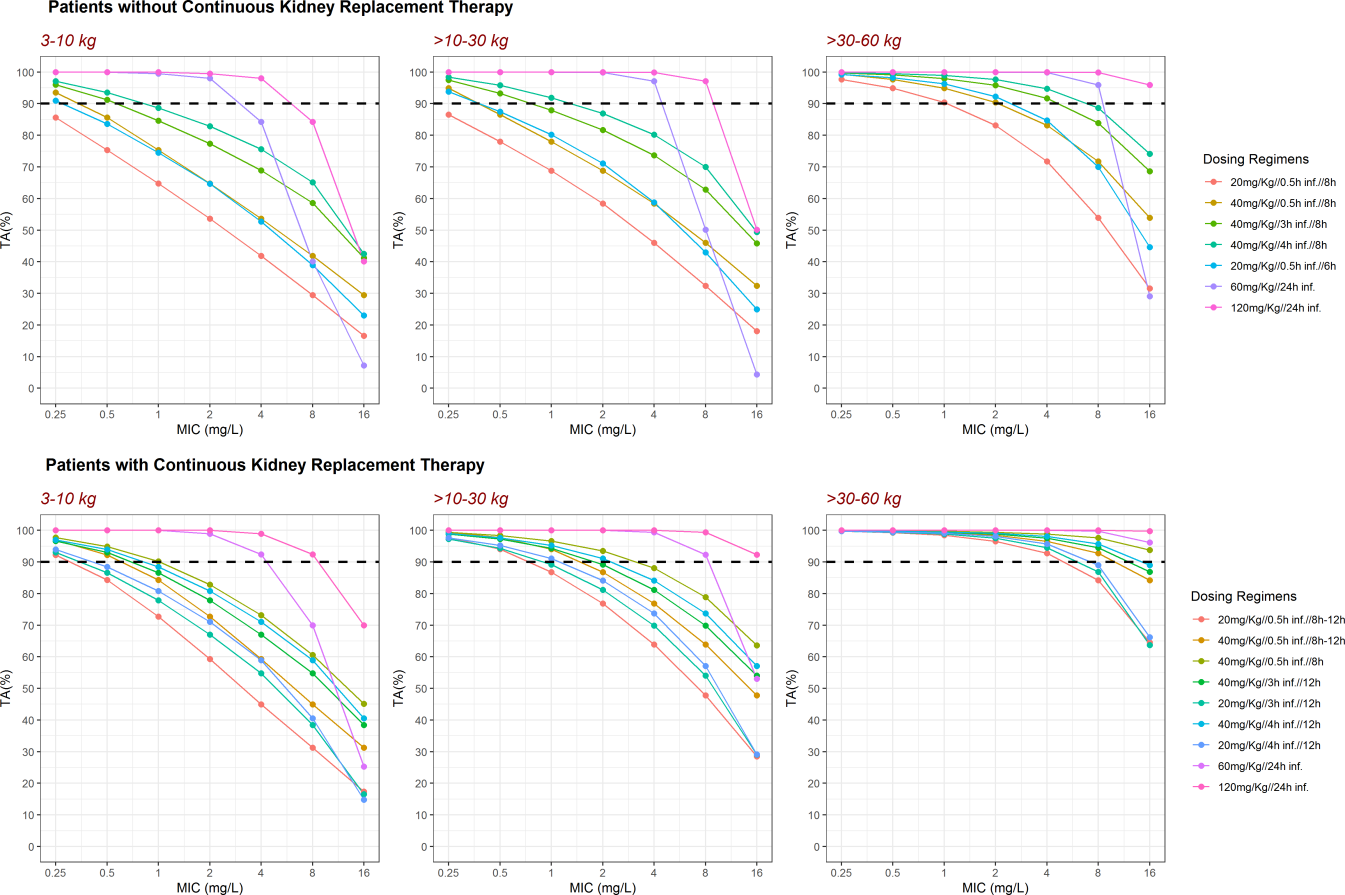
Target attainment. Each panel shows the percentage target attainment (%TA) per total body weight group for patients without (upper panels) and with (lower panels) continuous kidney replacement therapy. Each colored profile represents one of the dosing schedules listed on the right. %TA is calculated as the mean% time above minimum inhibitory concentrations during the first dosing interval of the second day of treatment.

Figure 4 and Fig. S2 compares the PTA of the current schedule with alternative dosing regimens in the absence or presence of a loading dose, respectively. For both CKRT and no-CKRT patients and weight groups, a continuous infusion of 60 mg/kg (CKRT) and 120 mg/kg (no-CKRT) every 24 h provides the best PTA across all values of MIC.

For patients without CKRT (simulation Fig. 4, graphics, top), the current schedules (20–40 mg/kg every 8 h in a short infusion of 30 min) were clearly insufficient to reach 90% fT > MIC (for an MIC of 2). We present the results of different dose simulations including extended infusions (40 mg/kg/3 or 4 h infusion/8 h) and decreased dosing interval (20 mg/kg/0.5 h infusion/6 h) but only continuous infusion (60 and 120 mg/kg/24-h infusion) reached the 90%TA in patients <30 kg.

For patients undergoing CKRT (simulation Fig. 4, graphics, bottom), the current schedule (40 mg/kg/12 h from day 2 in a short infusion of 30 min) was clearly insufficient to reach 90% fT > MIC (for a MIC >2) in patients <30 kg. Keeping the dose to 40 mg/kg q8h without applying renal adjustment and extended infusions (40 mg/kg/3 or 4-h infusion every 12 h) was appropriate to reach 90% fT > MIC (for an MIC of 2) in patients >10 kg. In patients <10 kg, only continuous infusions reached the therapeutic objective. In patients >30 kg, a 60 mg/kg/24-h infusion is adequate and does not reach toxicity levels (see below) and avoids accumulation during a longer treatment.

Figure S2 shows a comparison of different continuous infusion treatment schedules with a loading dose of a 40 mg/kg bolus. Some authors have proposed these dosing regimens with the aim of achieving rapid therapeutic drug concentrations and reaching immediate clinical response in patients with sepsis (33).

**Fig 5:**
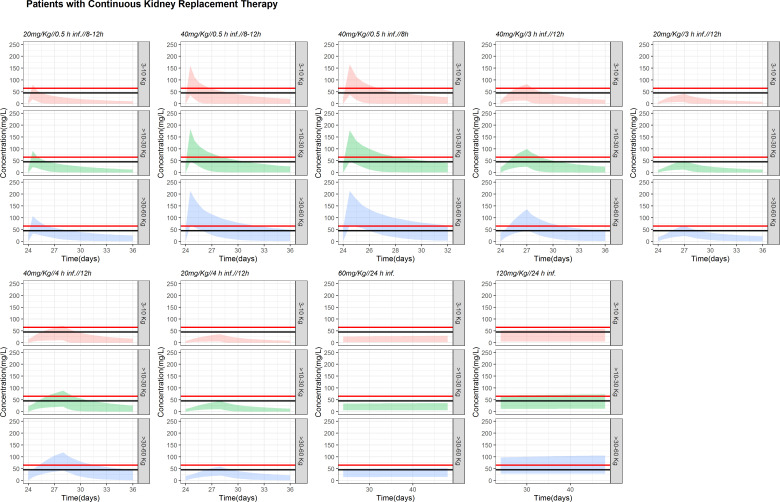
Full plasma concentration vs time profiles in patients with continuous renal replacement therapy. Colored areas cover the 95% prediction intervals of the concentrations generated from 1,000 virtual patients for each dosing scenario and weight group. The horizontal solid lines highlight the plasma values of 45 mg/mL (red) and 65 mg/mL (black).

Figure 5 and Fig. S4 contextualize the %TA vs MIC results considering toxicity levels. The black line (45 mg/L) and the red line (65 mg/L) represent the trough level to avoid nephrotoxicity and neurotoxicity, respectively (28). The predicted concentrations were below these limits, without evidence of accumulation, with the exception of patients higher than 30 kg with the high dose infusions (120 mg/kg/24 h) in patients with CKRT.

## DISCUSSION

In this work, the population-modeling approach has been applied to characterize the pharmacokinetic properties of the antibiotic meropenem in critically ill children with or without CKRT. Exposure of meropenem, measured in four different biological matrices, was well described over time in all patients. The analysis allowed the identification of three physiologically related covariates (total body weight, glomerular filtration rate, and postnatal age) and one related to the size of the hemofilter, affecting relevantly the distribution and elimination processes.

The proposed population model, which was extensively evaluated, provided precise typical and random effect parameters of a two-compartment disposition model. The selection of a multi-compartmental model is remarkable taking into account the reduced cohort of patient population and the sparse sampling design. Recently, Thy et al. (5) characterized the systemic disposition of meropenem in critically ill children with CKRT with the use of one-compartment model. It seems that there is evidence for a second/peripheral compartments based on the results from other analyses either in children (7) or in adults (34). Likely, in the present investigation, the limitation arising from the reduced number of patients and samples per patient was mitigated by the integrative and mechanistic approach adopted during the analysis.

The population parameter estimates for a typical individual without and with CKRTand total body weight, age, and eGFR of 10 kg, 42 months, and 95 mL/min/1.73 m^2^, respectively, were, in general, in agreement with those reported by other authors. For example, the total apparent volume of central compartment distribution and total clearances obtained by Saito et al. (7) were 4.9 L and 4.6 L/h, in agreement with the estimates listed in Table 4 (4.75 L and 3.93 L/h). Zyryanov et al. (35) developed a population pharmacokinetic model for meropenem in preterm newborns with mean body weight and mean gestational age of 1.66 kg and 28.9 weeks, respectively, reporting typical values of 1.24 L and 0.35 L/h, which are comparable to those in the present analysis with postnatal age of 2 weeks. Our results are also in accordance with those reported in adults. For example, the typical estimates of CL (L/h) provided by Peng et al. (34) and An et al. (36) were 3.03 and 5.28 (including non-renal clearance), respectively. The apparent volume of distributions ranged from 8.3 to 35 L (36, 37), which are expressed per kg of body weight, are similar to the value derived from Table 4 (0.45 L/kg). The typical estimate of the non-renal clearance represents 15% of the total elimination, according to the evidence that meropenem is primarily excreted by the kidney (38).

The estimates of the magnitude of the remaining unexplained interindividual variability in *V*1 (48%) and CL (30%) agree well with literature data (42%–58%, V1, and 47.1%–59.3 %, CL) (34, 36). The lower estimate of IIV on CL found in our case might be explained by the fact that the selected model considers the non-renal elimination route associated with elevated IIV (86%).

Most of the population pharmacokinetic analyses identified eGFR and body weight as significant covariates for CL and V1, respectively. The predicted percentage decreases in renal and total clearances for every reduction in 20 mL/min/1.73 m^2^ predicted by our model are 21% and 18%, respectively. Weight effects in CL for critically ill children receiving meropenem were reported by Rapp et al. and Saito et al. (7, 39). However, in the present evaluation, weight was not selected in the final model in favor of post-natal age, reflecting organ maturation beyond the value of eGFR. In fact, 90% of adult kidney functionality is predicted at ages over 12 months, corresponding to patients that might be considered adults and serving as the rationale for the lack of weight effects on CL in those analyses performed in the adult populations (34, 36).

Regarding the patients undergoing CKRT, our analyses indicated that for those patients that show diuresis, the estimate of renal clearance was five to sixfold lower than the corresponding value obtained in case of no CKRT (0.96 vs 5.4 L/h, respectively) and equal to the contribution of the non-renal elimination. The contribution of the extracorporeal represented 40% of the total clearance in patients with CKRT and using a filter of medium surface area. For patients with low and high surface area filters, the corresponding percentages were 15% and 78%, respectively. Interestingly, once the surface area was incorporated in the model as a covariate, the interindividual variability in CLCKRT vanished. A similar result was found in the case of piperacillin (40). It must be taken into consideration that meropenem concentrations in the effluent depend on CKRT parameters and also on the total effluent flow rate (*Q*_eff_), which is a variable that was controlled and known during the course of the treatment. The analysis did not find differences between patients with or without CKRT in the apparent volumes of distribution, inter-compartmental clearance, and CLM.

From the model published recently by Thy et al. (5), the obtained typical value of the apparent volume of distribution in a pediatric patient of 10 kg of body weight was 4.6 L, an estimate remarkably similar to the 4.5 L listed in Table 4. However, the integrative and mechanistic approach undertaken in the present analysis allowed us to characterize distribution to a peripheral compartment, and the resulting total apparent volume of distribution was 15.4 L. For the case of total clearance, an estimate of 1.34 L/h is associated with a patient with 10 kg of body weight, 4 years of age, and 1,200 mL/h of *Q*_eff_ based on the model selected by Thy et al. (5). That value is 37% and 56% lower than the corresponding values obtained from the current investigation in patients with CKRT and with or without diuresis. In the current model, neither body weight nor *Q*_eff_ was included as significant covariates of CL. In our patients, the values of *Q*_eff_ were highly correlated with body weight, which might explain differences between the two models, as *Q*_eff_ was included as a variable, which together with the estimate of CLCKRT predicts effluent concentrations.

Other models have been developed by measuring only prefilter concentrations (5, 39, 41). In our opinion, it is very important to simultaneously determine prefilter, postfilter, and effluent concentrations, as well as in the urine, when the patient has diuresis. This allows us to untangle the total elimination clearance into renal, non-renal, and the one representing the contribution of continuous kidney replacement therapy. We believe that the availability of meropenem concentrations in different biological matrices and the integrative analysis presented in this work have led to a better characterization of the disposition characteristics of meropenem in this critical patient population, allowing us to quantify variability in the distribution process.

When the CKRT technique is used early, not only in patients with severe kidney failure but also with the indication of avoiding positive balance, many patients maintain diuresis (42). Renal clearance may be relevant in these patients and the urine concentrations of these patients must be taken into account when developing a model.

Simulations of different potential dosing regimens paying attention to target attainment and plasma levels associated with toxicity have been performed with the selected model after an intense model evaluation and shown in Fig. 1.

Our results indicate that the standard dosing is associated with suboptimal target exposure. In this study, only 33% of the patients without CKRT achieved >90% time > MIC = 2 with the current dosing schedule. These results have already been described in other studies in adults and children (3, 43, 44) and could explain treatment failure in some of those patients. Moreover, subtherapeutical levels have been related with growing concern of multidrug-resistant infections (45). This led to the search for alternative dosing schemes to improve the PTA and the clinical outcome.

In previous studies in adults, continuous infusions achieved the pharmacodynamic targets more easily (46, 47). Also, the stability of meropenem at room temperature has been tested (48), and perfusion syringes must be changed every 8 h. However, administering intravenous continuous infusions in children is more difficult than in adults. In critically ill children, a central venous line is an ideal access to administer continuous infusions to avoid extravasation of the antibiotic (49). Drug-drug incompatibilities require the use of a separate infusion line for the antibiotic. Administering antibiotics through continuous infusions is not always possible due to the many drugs that a critically ill patient may require.

In this study, in children without CKRT receiving 40 mg/kg q8h in an extended 4-h infusion, the 90%TA was achieved for MIC ≤ 1 mg/L in all the weight groups and for MIC = 2 mg/L for patients >10 kg. This schedule represents a promising alternative to continuous infusions in case a separate IV line is not available. This alternative schedule has also been proposed in other pharmacokinetic studies in adults (50), and the first international recommendation for the use of extended infusions in adults was published in 2023 (51). Pediatric research on meropenem extended infusions is restricted to single-center retrospective clinical studies (52).

In patients with CKRT, the historically applied renal adjustment for meropenem fails to achieve adequate concentrations because CKRT removal of this antibiotic is relevant. As in Thy et al. (5), this is especially important in patients <10 kg. Thus, body weight has to be taken into account in renal adjustment when prescribing meropenem in these patients. Maintaining the dose to 40 mg/kg q8h without applying renal adjustment could be more appropriate in these types of patients. Retrospective data regarding this dosing strategy in children with CKRT and meropenem are not available, and therefore, drug monitoring is warranted in these patients.

To reach 90% fT > MIC (for an MIC of 2) in patients <10 kg, a 60–120 mg/kg continuous infusion is necessary. In patients >30 kg, a 60 mg/kg/24-h infusion is sufficient and could avoid accumulation in a prolonged treatment.

Although the relationship between plasma concentrations and clinical adverse effects is poorly described, previous studies in adults advise keeping the trough concentration between 16 [8× MIC 2 (26)] and a maximum of 45 mg/mL (to avoid nephrotoxicity) or 65 mg/mL [to avoid neurotoxicity (27, 28)]. However, more data are needed to establish the toxicity threshold in children.

### Limitations

The major limitation of this study is the reduced sample size. This was mitigated by the integrative and mechanistic approach adopted during the analysis. Simulations help to diminish the impact of these limitations and an external validation cohort would strengthen the current analysis. Also, neonatal patients, less than 28 days of postnatal age, were not included in this study. It should be considered that most patients (62.5%) of the group were in the postoperative period of cardiac surgery. Additionally, CKRT protocol and settings, such as the effluent flow, may not be equivalent between centers. A multicenter study could help to determine the effect of these differences.

### Conclusions

This analysis demonstrates that the clearance of meropenem by extracorporeal systems is relevant and should be integrated into pharmacokinetic predictions for critically ill pediatric patients. The standard pediatric dose of 20–40 mg/kg/8 h bolus could be insufficient to achieve optimal meropenem exposures in a significant proportion of critically ill children. In pediatric patients without AKI, a 60 mg/kg/24 h continuous infusion or, as an alternative in patients >10 kg, 40 mg/kg q8h in an extended 4-h infusion is necessary to treat bacteria with ≤MIC 2 mg/L for meropenem.

In pediatric patients undergoing CKRT, keeping the dose to 40 mg/kg q8h without applying renal adjustment and the extended infusions (40 mg/kg/3 or 4-h infusion every 12 h) was sufficient to reach 90% fT > MIC (for an MIC of 2 mg/L) in patients > 10 kg. In patients <10 kg, only continuous infusions reached the objective. In patients >30 kg, a 60 mg/kg/24-h infusion is sufficient and avoids accumulation.

Model-informed precision dosing must be the objective in this especially vulnerable population, which will be accomplished through dedicated population pharmacokinetic studies in children on CKRT. Combining these studies with individual TDM measures warrants improved personalized antibiotic management.

## SUPPLEMENTAL MATERIAL

Figure S1 aac.01729-23-s0001.tiff Body weight vs age relationship.

Figure S2 aac.01729-23-s0002.tiff Target attainment including loading dose.

Figure S3 aac.01729-23-s0003.tiff Full plasma concentration vs time profiles in patients without continuous renal replacement therapy.

Figure S4 aac.01729-23-s0004.tiff Full plasma concentration vs time profiles in patients with/without continuous renal replacement therapy including a loading dose.

Supplemental legends aac.01729-23-s0005.docx Legends for Fig. S1 to S4.

ASM does not own the copyrights to Supplemental Material that may be linked to, or accessed through, an article. The authors have granted ASM a non-exclusive, world-wide license to publish the Supplemental Material files. Please contact the corresponding author directly for reuse.
