# Supplementary figures and images for: How to use meropenem in pediatric patients undergoing CKRT? Integrated meropenem pharmacokinetic model for critically ill children
Source: Antimicrob Agents Chemother. 2024 Apr 24;68(6):e01729-23. doi: 10.1128/aac.01729-23 (PMC11620509; doi:10.1128/aac.01729-23)

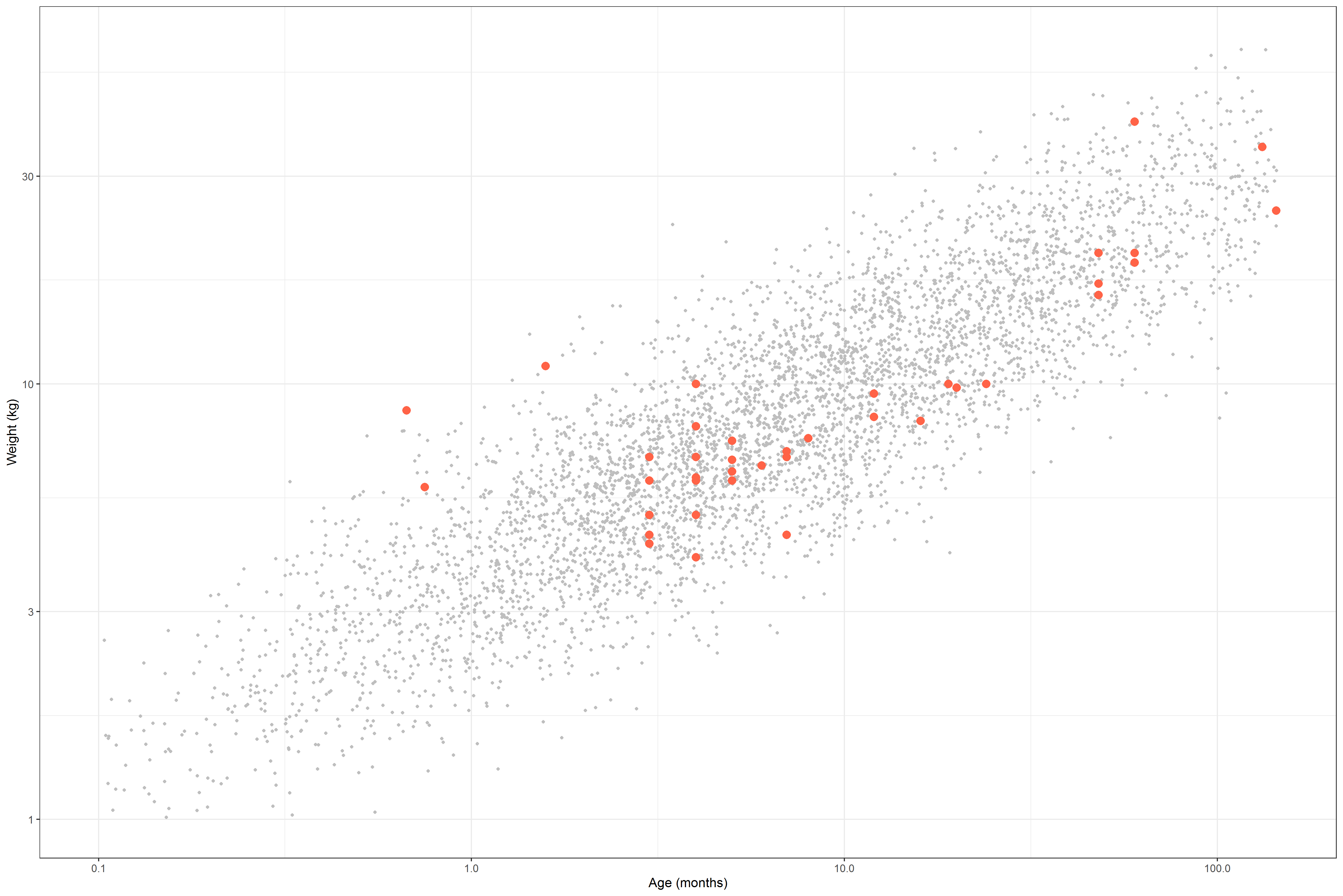

Supplement: Figure S1 — Body weight vs age relationship. [file aac.01729-23-s0001.tiff]

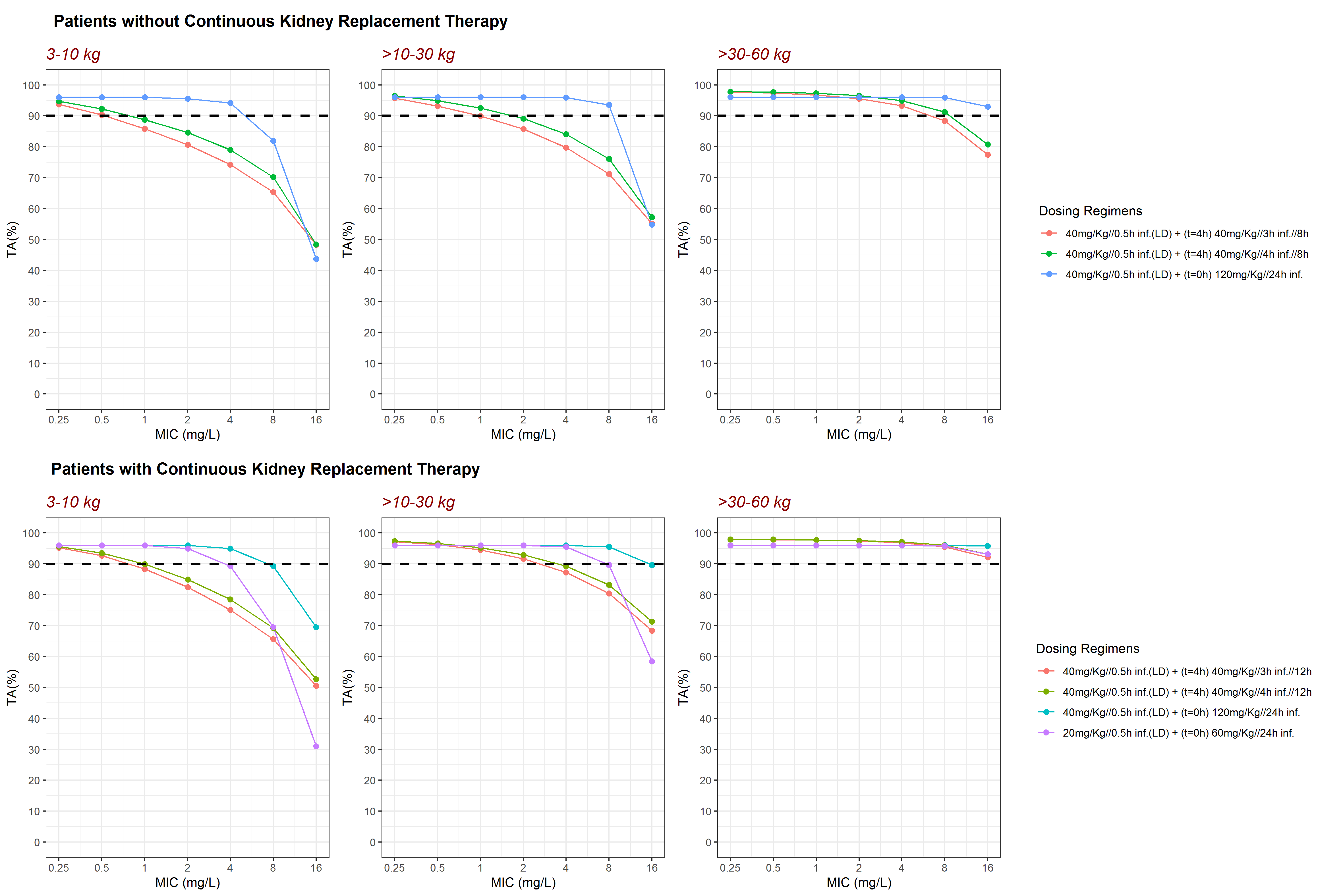

Supplement: Figure S2 — Target attainment including loading dose. [file aac.01729-23-s0002.tiff]

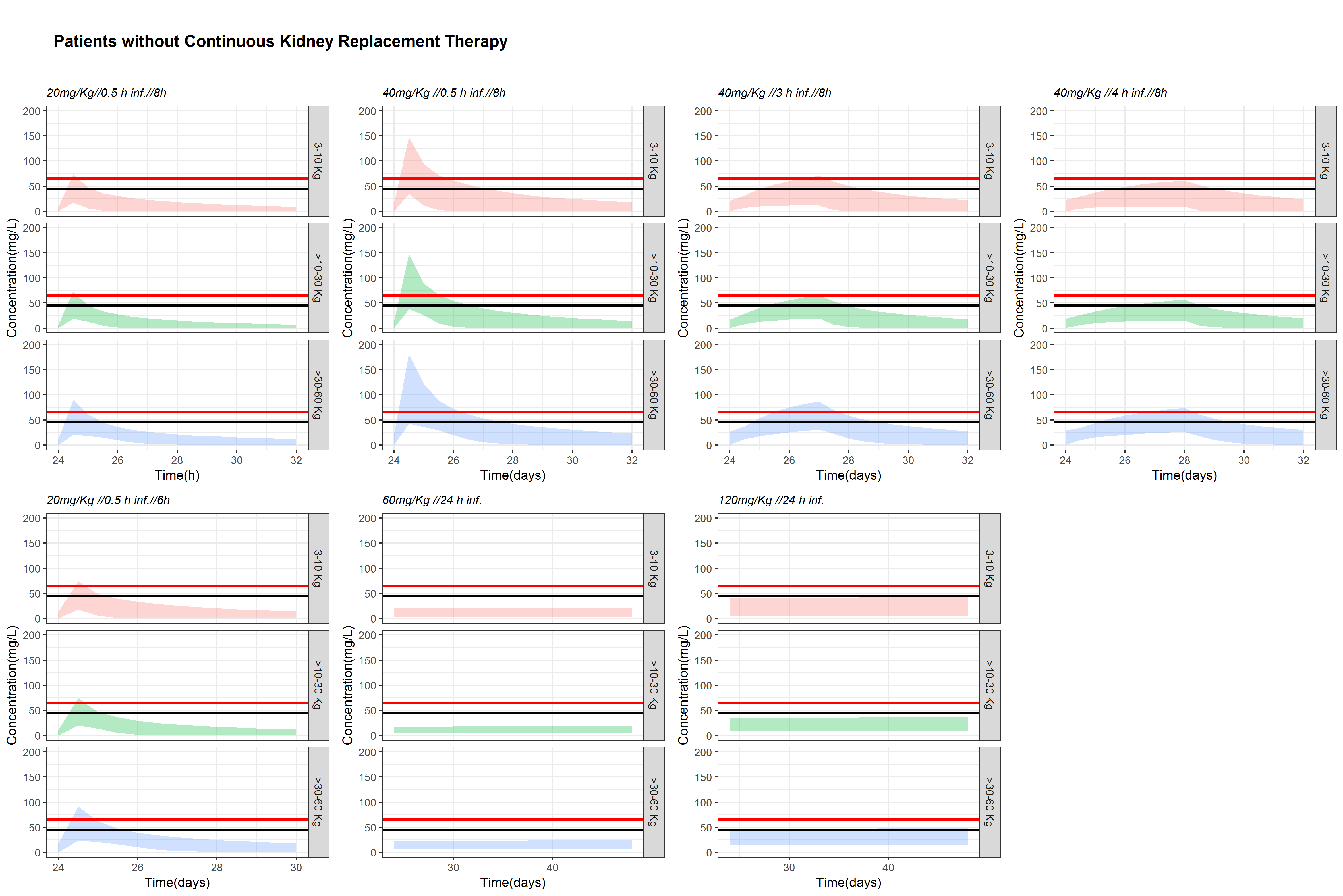

Supplement: Figure S3 — Full plasma concentration vs time profiles in patients without continuous renal replacement therapy. [file aac.01729-23-s0003.tiff]

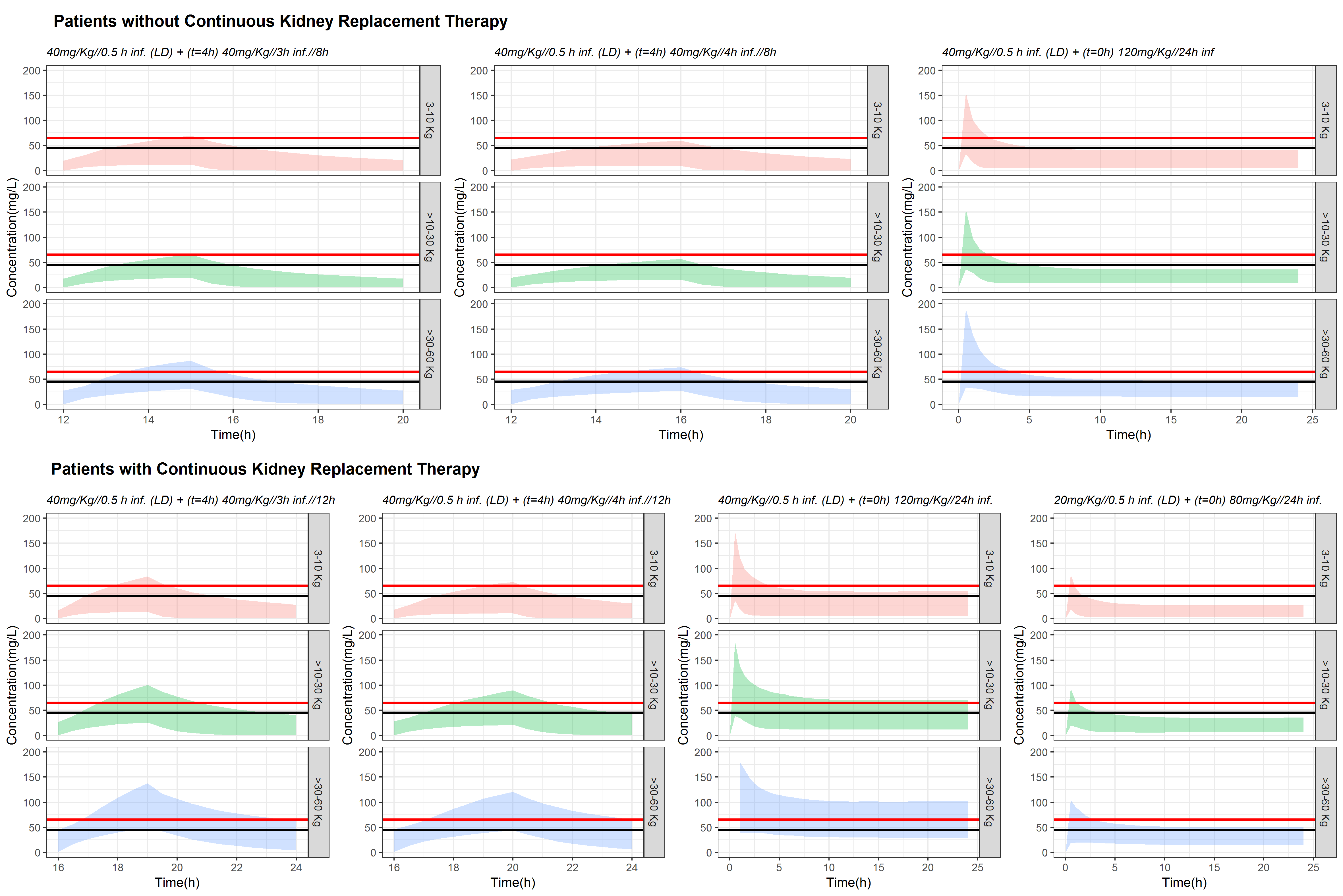

Supplement: Figure S4 — Full plasma concentration vs time profiles in patients with/without continuous renal replacement therapy including a loading dose. [file aac.01729-23-s0004.tiff]
