# Supplementary material for: How to use meropenem in pediatric patients undergoing CKRT? Integrated meropenem pharmacokinetic model for critically ill children
Source: Antimicrob Agents Chemother. 2024 Apr 24;68(6):e01729-23. doi: 10.1128/aac.01729-23 (PMC11620509; doi:10.1128/aac.01729-23)
Supplement: Supplemental legends — Legends for Fig. S1 to S4. [file aac.01729-23-s0005.docx]

**Legend to figures**

**Supplementary material/Figure 1.** ***Body weight vs age relationship.*** Points in grey represent five thousand simulated individual weight vs age pairs. Red circles correspond to values obtained from real patients.

**Supplementary material/Figure 2. *Target attainment including loading dose*.** Each panel shows the percentage target attainment (%TA) per total body weight group for patients without (*upper panels*) and with (*lower panels*) continuous kidney replacement therapy. Each colored profile represents one of the dosing schedules listed on the right ***which includes a loading dose (LD); the time in parenthesis corresponds to the initiation of the maintenance dosing schedule***. %TA is calculated as the mean % time above minimum inhibitory concentrations (MIC) during the first dosing interval of the second day of treatment.

**Supplementary material/Figure 3. *Full plasma concentration vs time profiles in patients without continuous renal replacement therapy.*** Colored areas cover the 95% prediction intervals of the concentrations generated from 1000 virtual patients for each dosing scenarios and weight group. The horizontal solid lines highlight the plasma values of 45 mg/mL (red) and 65 mg/mL (black).

**Supplementary material/Figure 4. *Full plasma concentration vs time profiles in patients with /without continuous renal replacement therapy including a loading dose.*** Colored areas cover the 95% prediction intervals of the concentrations generated from 1000 virtual patients for each dosing scenarios and weight group. The horizontal solid lines highlight the plasma values of 45 mg/mL (red) and 65 mg/mL (black).

**Table 1S.** Patient’s main diagnosis and infections treated. Causative bacteria and MIC when isolated. Patients sorted by group and age.

| Group | Age (months) | Main diagnosis | Infection | Isolated bacteria | MIC (mg/l) |
| --- | --- | --- | --- | --- | --- |
| **CKRT** | 3 | Congenital heart disease | Bacteriemia | Enterobacter aerogenes | MIC ≤ 0.12 |
|  | 5 | Congenital heart disease | ND | ET | - |
|  | 26 | Congenital heart disease | Septic shock | ET | - |
|  | 48 | Septic shock | Septic shock | ET | - |
|  | 60 | Congenital heart disease | ND | ET | - |
|  | 156 | Pediatric cerebral palsy  Scoliosis surgery | Septic shock  Pneumonia  Ecthyma gangrenosum | ET  P. aeruginosa  K. oxytoca  ET | MIC ≤1  MIC ≤1 |
|  | 168 | Refractory septic shock | Pneumonia | S. Aureus, pneumococo  (PCR assay in pleural effusion) | - |
| **NO CKRT** | 3 | Congenital heart disease | ND | ET | - |
|  | 3 | Congenital heart disease surgery | Bacteriemia | S. maltophilia  K. pneumoniae | MIC >32 R  MIC ≤ 0.12 |
|  | 4 | Congenital heart disease | Urinary tract infection | Klebsiella oxytoca  E. Coli | MIC ≤1  MIC ≤1 |
|  | 7 | Myocarditis | ND | ET | - |
|  | 9 | Congenital heart disease | Bacteriemia | Serratia marcescens | MIC ≤ 0.12 |
|  | 19 | Myocarditis | Urinary tract infection  Bacteriemia | Klebsiella pneumoniae | MIC ≤ 0.12 |
|  | 20 | Esophageal atresia and tracheoesophageal fistula | Pneumonia | ET | - |
|  | 144 | Congenital heart disease | Pneumonia | Klebsiella pneumoniae | MIC ≤1 |
|  | 173 | Congenital heart disease surgery | Bacteriemia | P. aeruginosa | MIC ≤ 0.12 |

ND: Not determined (fever, clinical deterioration ± acute phase reactants elevation) without microorganism isolation. ET: Empiric antimicrobial therapy.
